# Association between retinopathy and risk of dementia in a general Japanese population: the Hisayama Study

**DOI:** 10.1038/s41598-024-62688-7

**Published:** 2024-05-26

**Authors:** Shun Nakamura, Emi Ueda, Tomoyuki Ohara, Jun Hata, Takanori Honda, Kohta Fujiwara, Yoshihiko Furuta, Mao Shibata, Sawako Hashimoto, Taro Nakazawa, Tomohiro Nakao, Takanari Kitazono, Koh-Hei Sonoda, Toshiharu Ninomiya

**Affiliations:** 1 Department of Epidemiology and Public Health, Graduate School of Medical Sciences, Kyushu University, Fukuoka, Japan; 2 Department of Ophthalmology, Graduate School of Medical Sciences, Kyushu University, 3-1-1 Maidashi, Higashi-Ku, Fukuoka, 812-8582 Japan; 3 Department of Neuropsychiatry, Graduate School of Medical Sciences, Kyushu University, Fukuoka, Japan; 4 Center for Cohort Studies, Graduate School of Medical Sciences, Kyushu University, Fukuoka, Japan; 5 Department of Medicine and Clinical Science, Graduate School of Medical Sciences, Kyushu University, Fukuoka, Japan; 6 Department of Psychosomatic Medicine, Graduate School of Medical Sciences, Kyushu University, Fukuoka, Japan

**Keywords:** Risk factors, Predictive markers, Cognitive ageing, Cognitive neuroscience, Retina

## Abstract

We investigated the association of retinopathy with the risk of dementia in a general older Japanese population. A total of 1709 population-based residents aged 60 years or older without dementia were followed prospectively for 10 years (2007–2017). They underwent color fundus photography in 2007. Retinopathy was graded according to the Modified Airlie House Classification. Main outcome was the Incidence of dementia. A Cox proportional hazards model was used to estimate the hazard ratios (HRs) and their 95% confidence intervals (CIs) for the risk of dementia by the presence of retinopathy. During the follow-up period, 374 participants developed all-cause dementia. The cumulative incidence of dementia was significantly higher in those with retinopathy than those without (*p* < 0.05). Individuals with retinopathy had significantly higher risk of developing dementia than those without after adjustment for potential confounding factors (HR 1.64, 95% CI 1.19–2.25). Regarding the components of retinopathy, the presence of microaneurysms was significantly associated with a higher multivariable-adjusted HR for incident dementia (HR 1.94, 95% CI 1.37–2.74). Our findings suggest that, in addition to systemic risk factors, retinal microvascular signs from fundus photography provide valuable information for estimating the risk of developing dementia.

## Introduction

Dementia is a worldwide priority both in terms of public health and social care because of the rapidly increasing burdens it places on communities^1^. Neuroimaging examinations have been useful for directly detecting neuropathology related to dementia, but they are limited to highly specialized clinics or hospitals with cost-intensive equipment, and are unlikely to be widely available^2,3^. The retina and the brain have similar anatomical structures, physiological characteristics, and embryological origins^4^. Clinical studies have suggested that retinal involvement is associated with the presence of cognitive impairment^5–7^.

Retinopathy is known to be associated with small vessel disease in the retina, not limited to specific causes such as diabetes or hypertension^8–10^. The signs of retinopathy consist of microaneurysms, blot- or flame-shaped hemorrhages, and hard or soft exudates^11^, which can be visualized noninvasively by retinal fundus photography. Previous pathological studies of dementia have reported disorders of the small blood vessels in both the retina and brain of patients with dementia^4,12–14^. In addition, population-based neuroimaging studies have shown that retinal microvascular abnormalities are associated with the prevalence and progression of brain microvascular diseases such as subclinical brain infarction and white matter lesions^15,16^. These findings raise the possibility of an association between retinal microvascular abnormalities and the risk of incident dementia. However, there have been few population-based longitudinal studies addressing this issue, and the results from those studies have been inconsistent^17,18^. Therefore, further population-based studies on the association between retinopathy and the development of dementia are warranted.

The Hisayama Study is an ongoing, population-based prospective cohort study of non-communicable diseases including cardiovascular disease, dementia, and eye disease in a Japanese community^19^. The present study sought to investigate the association between retinopathy and risk for the development of dementia by using fundus photograph data and prospective cohort data of dementia patients in a general older Japanese population.

## Methods

### Study population

The Hisayama Study has been conducted since 1961 in the Hisayama town, a suburban community adjacent to the city of Fukuoka in Japan^20^. Since 1985, we have also conducted comprehensive surveys of dementia every 5–7 years (i.e., 1985, 1992, 1998, 2005, 2012, 2017, and 2022) in older residents of this town to identify individuals with dementia or disability in activities of daily living^21,22^. In addition, a survey of eye diseases among residents of this town has been under way since 1998^23^.

In 2007 and 2008, a screening survey for the present study was performed in the town. A total of 2002 residents ≥ 60 years of age (86.3% of the total population of this age group) consented to participate in the examination. Of these, we excluded 6 individuals who did not provide their consent for study participation, 180 individuals who had already developed dementia at baseline, 105 individuals who did not undergo a screening for eye disease, and 2 individuals with poor-quality fundus photographs. After exclusion of these cases, a total of 1709 participants (750 men and 959 women) were enrolled in the present study.

### Diagnosis of retinopathy

Each participant underwent an ophthalmic examination, during which their pupils were dilated using a solution containing 1.0% tropicamide and 10% phenylephrine. Subsequently, nonstereoscopic fundus photographs with a field angle of 45° were captured utilizing a digital fundus camera (TRC NW-6SF; Topcon Corporation). Specifically, one field was photographed for each eye, and this field was centered at a point equidistant between the temporal edge of the optic disc and the fovea. All obtained fundus photographs were subject to independent evaluation by two seasoned ophthalmologists, namely S.N. and E.U. In cases where there was a discrepancy in their assessments, the photographs were subjected to further examination by a panel of three retinal specialists, namely S.N., E.U., and K.S. A final judgment was reached through comprehensive discussion among these experts. The presence of retinal lesions was defined as a level of 15 or greater based on the modified Airlie House Classification: that is, the presence of any of the following lesions—microaneurysms, blot- or flame-shaped hemorrhages, hard exudates, cotton wool spots—or evidence of laser treatment for retinopathy. The graders judged retinal findings from fundus photographs without assumption of cause by masking systemic information such as hypertension and diabetes^11,24^.

### Follow-up surveys of dementia

Participants in the study were monitored prospectively from the commencement of a baseline examination in 2007–2008 until November 30, 2017 (with a median duration of 10.2 years and an interquartile range of 9.3–10.4 years). Details of the follow-up survey regarding dementia have been previously documented^22^. In summary, the identification of new dementia cases was achieved through a daily monitoring system that was jointly managed by the study team, local physicians, and members of the town's Health Office. This system involved regular visits by study team physicians to clinics, hospitals, and the town's office to gather information on dementia or stroke events, including suspected cases. Additionally, annual health check-ups, which included both physical and neurological examinations, were conducted to capture any incidents of dementia that may have been missed by the monitoring system. For individuals who did not undergo regular examinations or had relocated from the town, their health information was verified annually through correspondence or phone contact. If a participant displayed signs of new neurological symptoms, particularly those suggestive of dementia, they underwent a thorough evaluation by expert psychiatrists and stroke physicians from the study team. Furthermore, in 2012 and 2017, comprehensive assessments of cognitive function, including neuropsychological tests like the Mini-Mental State Examination^25^, were administered to maximize the accurate detection of dementia cases. In the unfortunate event of a participant's passing, a meticulous review was conducted, which encompassed gathering and analyzing all accessible medical records, including neuroimaging (CT/MRI), and conducting interviews with the deceased's family and attending physician. Throughout the follow-up period, there were 384 participant deaths, and autopsies were performed on 241 of these individuals. Importantly, no participants were lost to follow-up due to a lack of survival or dementia event information.

### Diagnosis of dementia

The diagnosis of dementia in this study adhered to the criteria outlined in the Diagnostic and Statistical Manual of Mental Disorders, 3rd edition, revised (DSM-III-R)^26^. To specify further, the criteria of the National Institute of Neurological and Communicative Disorders and Stroke and the Alzheimer’s Disease and Related Disorders Association (NINCDS-ADRDA)^27^ were utilized for diagnosing Alzheimer’s disease (AD), while the criteria of the National Institute of Neurological Disorders and Stroke-Association International pour la Recherche et l’Enseignement en Neurosciences (NINDS-AIREN) were employed for the diagnosis of vascular dementia (VaD)^28^. The determination of probable or possible subtypes of dementia was established based on a comprehensive evaluation of clinical data and the analysis of neuroimaging findings. For participants who underwent autopsy and were diagnosed with dementia, we conducted a thorough examination, combining neuropathological findings with clinical data, to arrive at definitive subtypes of dementia. A detailed description of the diagnostic process for autopsied cases with dementia has been previously documented^29^. In every instance where a participant was suspected of having dementia or in cases of participant demise during the follow-up period, a panel of expert psychiatrists and stroke physicians meticulously reviewed all available medical information. This thorough assessment was conducted to confirm the presence or absence of dementia and to ascertain the specific subtype of dementia when applicable.

### Measurements of covariates

Each participant completed a self-administered questionnaire that encompassed various aspects of their medical history and lifestyle. The questionnaire covered information regarding medical treatment (including medications for hypertension and diabetes mellitus), educational background, smoking habits, alcohol consumption, and regular physical activity. Low educational status was defined as having received ≤ 9 years of formal education. Smoking habits and alcohol consumption were categorized as either current and habitual or non-habitual. To determine regular exercise, participants were identified as such if they engaged in any form of physical activity three or more times per week during their leisure time. Blood pressure was measured three times using an automated sphygmomanometer, following more than five minutes of rest in a seated position. The analysis utilized the mean of these three measurements. Hypertension was diagnosed when blood pressure levels were ≥ 140/90 mmHg and/or when participants were currently taking antihypertensive medications. Plasma glucose levels were assessed using the hexokinase method. Diabetes mellitus was defined in the following manner: fasting glucose levels ≥ 7.0 mmol/L, casual or 2-h post-load glucose levels ≥ 11.1 mmol/L, and/or the use of glucose-lowering medications. Serum total cholesterol levels were enzymatically determined. Body mass index (BMI) was computed by dividing body weight in kilograms by the square of height in meters. These measurements were taken with participants wearing light clothing and no shoes. History of stroke was ascertained based on the presence of a documented sudden onset of non-convulsive and focal neurological deficits lasting for > 24 h. This determination was made by considering all available clinical data, which included medical records, neurological examinations, and brain imaging.

### Statistical analysis

Mean values or frequencies of risk factors across the presence or absence of retinopathy were estimated and tested by linear or logistic regression analysis, respectively. The cumulative incidence of all-cause dementia across the presence or absence of retinopathy was calculated by the Kaplan–Meier method and compared by the log-rank test. The Cox proportional hazards model was used to estimate the age- and sex-adjusted and multivariable-adjusted hazard ratios (HRs) and their 95% confidence intervals (CIs) for the development of all-cause dementia and its subtypes according to the presence or absence of retinopathy. The multivariable-adjusted model included age, sex, educational level, systolic blood pressure, antihypertensive medication, diabetes mellitus, serum total cholesterol, body mass index, history of stroke, smoking habits, alcohol intake, and regular exercise. All statistical analyses were performed with SAS, version 9.4 (SAS Institute, Cary, NC). Two-sided values of *p* < 0.05 were considered statistically significant.

### Ethical considerations

This study was approved by the Kyushu University Institutional Review Board for clinical research (2022-151) and was carried out in accordance with the Declaration of Helsinki and Ethical Guidelines for Medical and Biological Research Involving Human Subjects in Japan (https://www.lifescience.mext.go.jp/files/pdf/n2312_01.pdf [Japanese]). Informed consent was obtained from all participants.

## Results

Among the 1709 study participants, 174 participants (10.2%) had retinopathy. The baseline characteristics of the study population by the presence or absence of retinopathy are shown in Table 1. The mean systolic blood pressure, diastolic blood pressure, and body mass index and the proportions of male subjects, use of antihypertensive agents, diabetes mellitus, and history of stroke were significantly higher in the individuals with retinopathy compared to those without, whereas the mean serum total cholesterol levels were significantly lower in the participants with retinopathy than those without.

**Table 1:** Baseline characteristics of subjects according to the presence or absence of retinopathy: the Hisayama Study, 2007.

Variable	Retinopathy		*p* value
	Absence (n = 1535)	Presence (n = 174)	
Age, mean (SD), year	71.3 (7.6)	71.8 (7.5)	0.40
Male, %	42.9	47.7	0.02
Education ≤ 9 years, %	43.9	42.0	0.64
Systolic blood pressure, mean (SD), mmHg	135 (18)	142 (19)	< 0.001
Diastolic blood pressure, mean (SD), mmHg	79 (10)	82 (10)	< 0.001
Use of antihypertensive agents, %	41.9	58.1	< 0.001
Hypertension, %	59.2	79.9	< 0.001
Fasting blood glucose, mean (SD), mmHg	105 (19)	118 (33)	< 0.001
Casual or 2-h postload glucose, mean (SD), mmHg	150 (65)	205 (110)	< 0.001
Use of glucose-lowering agents, %	8.2	33.7	< 0.001
Diabetes mellitus, %	18.0	42.2	< 0.001
Serum total cholesterol, mean (SD), mmol/L	5.39 (0.91)	5.14 (0.89)	< 0.001
Body mass index, mean (SD), kg/m^2^	23.0 (3.4)	23.6 (3.4)	0.03
History of stroke, %	4.8	9.8	0.007
Smoking habits, %	13.9	11.5	0.39
Alcohol intake, %	41.6	45.4	0.33
Regular exercise ≥ 3 times/w, %	13.9	13.2	0.81

*SD* standard deviation.

All values are given as the mean or as a percentage.

**Figure 1:**
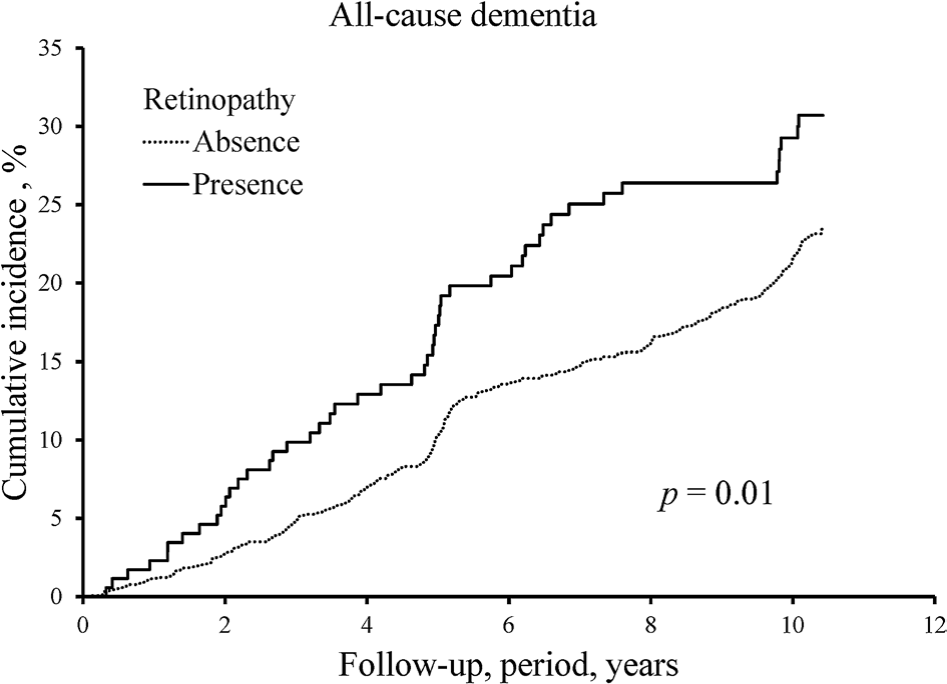
The cumulative incidence of all-cause dementia according to the presence or absence of retinopathy: the Hisayama Study, 2007–2017. The horizontal axis shows the follow-up period and the vertical axis shows the cumulative incidence of all-cause dementia.

During the 10-year follow-up period, 374 participants (136 men and 238 women) developed all-cause dementia. Of these, 264 participants experienced AD and 66 participants experienced VaD. Figure 1 demonstrates the cumulative incidences of all-cause dementia according to the presence or absence of retinopathy. The cumulative incidence of all-cause dementia was significantly higher in the participants with retinopathy compared with those without (*p* < 0.05). Table 2 shows the estimated HRs and 95% CIs for the development of all-cause dementia according to the presence or absence of retinopathy. The age- and sex-adjusted risk of all-cause dementia was significantly higher in the participants with retinopathy compared with those without retinopathy as a reference group (HR 1.56, 95% CI 1.15–2.11, *p* = 0.004). These associations were essentially unchanged even after additional adjustment for potential confounding factors, such as education level, systolic blood pressure, use of antihypertensive agents, serum total cholesterol, body mass index, diabetes mellitus, history of stroke, smoking habits, alcohol intake, and regular exercise (HR 1.64, 95% CI 1.19–2.25, *p* = 0.002). With regard to the subtypes of dementia, the presence of retinopathy was significantly associated with a higher risk for the development of AD (HR 1.50, 95% CI 1.01–2.23, *p* = 0.04), and marginally associated with a higher risk for the development of VaD (HR 1.84, 95% CI 0.93–3.62, *p* = 0.08) after adjustment for potential confounding factors in Supplementary Table. We also conducted a sensitivity analysis after excluding participants who developed dementia within the first 2 years of follow-up, and the result was substantially unchanged in Supplementary Table 2.

**Table 2:** Hazard ratios for the development of all-cause dementia according to the presence or absence of retinopathy and components of retinopathy: the Hisayama Study, 2007–2017.

	No. of events	No. at risk	Age- and sex-adjusted			Multivariable-adjusted^a^		
			HR	95% CI	*p* value	HR	95% CI	*p* value
Retinopathy								
Absence	325	1535	1.00	Reference		1.00	Reference	
Presence	49	174	1.56	1.15–2.11	0.004	1.64	1.19–2.25	0.002
Microaneurysm								
Absence	334	1570	1.00	Reference		1.00	Reference	
Presence	40	139	1.77	1.27–2.47	< 0.001	1.94	1.37–2.74	< 0.001
Retinal hemorrhage								
Absence	345	1598	1.00	Reference		1.00	Reference	
Presence	29	111	1.30	0.89–1.90	0.18	1.32	0.89–1.97	0.17

*CI* confidence interval; *HR* hazard ratio.

^a^Adjusted for age, sex, education, systolic blood pressure, use of antihypertensive agents, diabetes mellitus, serum total cholesterol, body mass index, history of stroke, smoking habits, alcohol intake, and regular exercise.

Of 174 participants with retinopathy, 139 (8.1%) had microaneurysm, 111 (6.5%) had retinal hemorrhage, 16 (0.9%) had hard exudate, and 6 (0.4%) had soft exudate. As shown in Table 2, the multivariable-adjusted HR for all-cause dementia was significantly higher in the individuals with microaneurysm than those without (HR 1.94, 95% CI 1.37–2.74, *p* < 0.001), whereas there was no evidence of significant association between the presence of retinal hemorrhage and dementia risk (HR 1.32, 95% CI 0.89–1.97, *p* = 0.17). The associations of hard exudate and soft exudate with the risk of the development of dementia could not be assessed due to the small number of participants with these retinal lesions in the present study.

**Figure 2:**
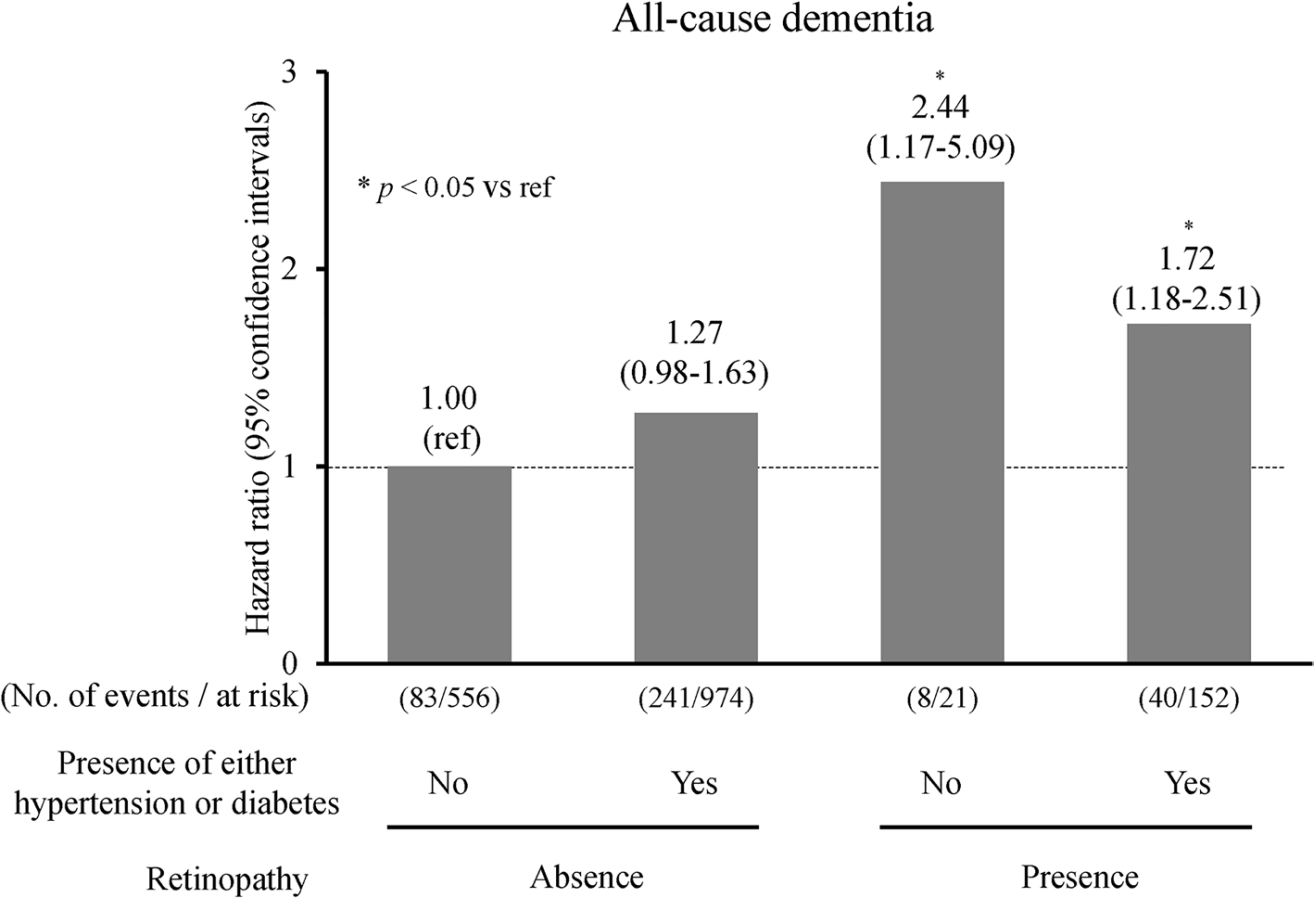
The combined influence of retinopathy and hypertension and/or diabetes mellitus on the development of all-cause dementia: the Hisayama Study, 2007–2017. The horizontal axis of the graph shows the combination of the presence of hypertension and/or diabetes mellitus and the presence of retinopathy, the number of participants who developed dementia and the total number of participants. The vertical axis of the graph shows the age- and sex-adjusted HR of the development of dementia. The corresponding HRs and 95% CI are shown above each bar. Adjusted for age and sex. *p < 0.05 vs reference. *HR* hazard ratio; *CI* confidence interval.

Furthermore, we investigated the combined associations of the presence of retinopathy and the presence of its affecting disease (i.e., hypertension or diabetes) with dementia risk, as hypertension and diabetes are both known to be systemic risk factors for retinopathy. As shown in Fig. 2, the age- and sex-adjusted HR for incident dementia increased significantly with the presence of retinopathy in those with either hypertension or diabetes (HR 1.72, 95% CI 1.18–2.51, *p* = 0.005). The risk of dementia increased significantly also among individuals with neither hypertension nor diabetes mellitus (HR 2.44, 95% CI 1.17–5.09, *p* = 0.02).

## Discussion

The present study clearly demonstrated that the risk of dementia increased significantly with the presence of retinopathy estimated by using fundus photography based on 10-year prospective data from a general older Japanese population. With regard to the components of retinopathy, the presence of microaneurysm was significantly associated with a higher risk of the development of dementia. Furthermore, the present study found that individuals with retinopathy had a significantly increased risk of incident dementia even if they did not have hypertension and diabetes mellitus. These findings suggested that, in addition to systemic risk factors of dementia, retinal microvascular signs from fundus photography might themselves provide valuable information for estimating the future risk of developing dementia.

Our results using the prospective data from a general Japanese population showed that the presence of retinopathy was significantly associated with a higher risk of the development of dementia in the study population. To our knowledge, only 2 population-based longitudinal studies have examined the association between retinopathy and the risk of dementia and their findings were inconsistent. Our findings were consistent with a report of the Atherosclerosis Risk in Communities Study, which showed that individuals with retinopathy had a significantly greater risk of dementia than those without^18^. On the other hand, the Rotterdam Study failed to reveal a significant association between retinopathy and the development of dementia^17,30^. This discrepancy in the findings among the studies may be attributable to the differences in the study design or population or the definition of retinopathy.

Some possible mechanisms that could explain the association between retinopathy and the development of dementia might exist. First, the presence of retinopathy may be a marker of the accumulation of systemic vascular risk factors, including hypertension and diabetes mellitus. These accumulated risk factors are well recognized as major risk factors of retinopathy^31^, as well as dementia^19,32,33^. Thus, an accumulation of vascular disorders such as atherosclerosis and abnormal glucose metabolism may influence this association. Second, the retinal microvascular abnormalities including microaneurysm may reflect the consequences of subclinical pathogenesis of dementia in the brain because the retina shares similar embryological origin, anatomical features and physiological properties with the brain^4^. Pathological studies have reported that the dysfunction of the vascular endothelial cells and blood barrier causes the change in both the retina and brain because of the common hemodynamic system^9,10,12–14^. Population-based neuroimaging studies^15,16^ have shown that retinal microvascular abnormalities are associated with the prevalence and progression of brain microvascular disease, including subclinical brain infarction and white matter lesions, which are known as a risk factor for incident dementia^2,3^. Taken together, the retinal microvascular signs may reflect similar pathophysiology to systemic vascular disorders and the cerebral microvasculature damage, which may be involved in the risk of dementia.

The present study showed that the risk of dementia increased even among individuals who had retinopathy but neither hypertension nor diabetes mellitus. Because of the limited sample size, it cannot be ruled out that the observed association merely reflects the play of chance. However, our findings in individuals without either hypertension or diabetes mellitus may be supported by some previous studies. The Atherosclerosis Risk in Communities Study also reported that retinopathy was associated with a greater decline of cognitive function among individuals without hypertension or diabetes mellitus^30,34^. Some population-based studies have described that approximately 1–5% of people without hypertension and/or diabetes had signs of retinal abnormalities, and these signs appeared to be aging^35,36^. In the present study, the individuals with retinopathy were likely to be older than those without hypertension and diabetes mellitus, although there was no evidence of a significant difference in other risk factors between individuals with and without retinopathy in Supplementary Table S3. The pathological studies suggested that both the retinal^37,38^ and the cerebral^39,40^ microvascular structures may occur in similar changes with aging. Together with these previous studies, the significant association in individuals without either hypertension or diabetes may reflect age-related changes in the microvascular structure.

With regard to the subtypes of dementia, the presence of retinopathy was significantly associated with a higher risk for the development of AD. The exact mechanisms underlying this association remain unclear, but the cerebral neurodegenerative changes leading to AD may result in similar pathological changes in the retinal microvasculature. Decreased retinal circulation has been reported in studies of patients with AD^41^. Neurotoxicity due to the accumulation of amyloid-β in the retinal vasculature may also cause damage to vascular epithelial cells, leading to retinal microvascular damage^42^. Meanwhile, the present study demonstrated that the presence of retinopathy was likely to have a higher risk for the development of VaD, but this association was not statistically significant, probably due to the limited event cases. Further prospective studies will be needed to clarify the issue related to the subtypes of dementia.

There are potential limitations that should be noted. First, the present study used one retinal examination at baseline, but not during the follow-up period. This may have led to some misclassification in the presence or absence of retinopathy. Second, we were unable to exclude the possibility that participants with prodromal dementia were included at baseline in the present analysis. Nonetheless, the inclusion of such participants would not have affected our main findings, since an excess risk of dementia was still observed in individuals with retinopathy in the sensitivity analyses after excluding dementia cases or death occurring within the first 2 years of follow-up in Supplementary Table 2. Third, we could not perform a detailed analysis regarding the association between the retinopathy and the subtypes of dementia or the association between each component of retinopathy and the dementia risk because of the limited sample size. Fourth, the generalizability of our findings may be limited, because the present study was conducted in only one community in Japan. The incidence rate in this study tended to be higher than in previous studies, and this might be due to the relatively older participants in this study, the difference in the survey method of dementia and the difference of race. Therefore, the findings of this study should be validated in other regions and countries.

In conclusion, the analysis of prospective longitudinal data from a general older population of Japanese revealed that the presence of retinopathy was significantly associated with the development of dementia in the study population. Our findings highlight the idea that the assessment of retinopathy by fundus photographs, which can noninvasively and conveniently visualize microvascular signs in the eye, might provide useful information for identifying high-risk individuals.

### Supplementary Information

Supplementary Information.

## Data Availability

The datasets generated and analyzed in the present study are not publicly available because they contain confidential clinical and demographic data of the study participants. However, further information about the datasets is available with the permission of the principal investigator of the Hisayama Study (Toshiharu Ninomiya) on reasonable request for purposes of replicating procedures and results.

## Abbreviations

AD: Alzheimer’s disease
VaD: Vascular dementia
HR: Hazard ratio
CI: Confidence interval
