# Supplementary material for: Association between retinopathy and risk of dementia in a general Japanese population: the Hisayama Study
Source: Sci Rep. 2024 May 26;14:12017. doi: 10.1038/s41598-024-62688-7 (PMC11128440; doi:10.1038/s41598-024-62688-7)
Supplement: Supplementary file 1 — Supplementary Information. [file 41598_2024_62688_MOESM1_ESM.docx]

**Supplementary Online Content**

eTable 1. Hazard ratios for the development of Alzheimer’s disease and vascular dementia according to the presence or absence of retinopathy.

eTable 2. Hazard ratios for the development of all-cause dementia and its subtypes according to the presence or absence of retinopathy when participants with dementia cases or death within the first 2 years were excluded.

eTable 3.　Baseline characteristics of participants according to the presence and absence of retinopathy among individuals without hypertension nor diabetes.

| **eTable 1. Hazard ratios for the development of Alzheimer’s disease and** **vascular dementia according to the presence or absence of retinopathy: the Hisayama Study, 2007-2017.** | | | | | |
| --- | --- | --- | --- | --- | --- |
|  | No. of events | No. at risk | Multivariable-adjusted ^a)^ | | |
|  |  |  | HR | 95% CI | *p* value |
| ***Alzheimer’s disease*** |  |  |  |  |  |
| Retinopathy |  |  |  |  |  |
| Absence | 234 | 1,535 | 1.00 | Reference |  |
| Presence | 30 | 174 | 1.50 | 1.01-2.23 | 0.04 |
| ***Vascular dementia*** |  |  |  |  |  |
| Retinopathy |  |  |  |  |  |
| Absence | 55 | 1,535 | 1.00 | Reference |  |
| Presence | 11 | 174 | 1.84 | 0.93-3.62 | 0.08 |
| Abbreviations: CI, confidence interval; HR, hazard ratio.  ^a)^ Adjusted for age, sex, education, systolic blood pressure, use of antihypertensive agents, diabetes mellitus, serum total cholesterol, body mass index, history of stroke, smoking habits, alcohol intake, and regular exercise. | | | | | |

| **eTable 2. Hazard ratios for the development of all-cause dementia and its subtypes according to the presence or absence of retinopathy when participants with dementia cases or death within the first 2 years were excluded: the Hisayama Study, 2007–2017.** | | | | | |
| --- | --- | --- | --- | --- | --- |
|  | No. of events | No. at risk | Multivariable-adjusted ^a)^ | | |
|  |  |  | HR | 95% CI | *p* value |
| ***All-cause dementia*** |  |  |  |  |  |
| Retinopathy |  |  |  |  |  |
| Absence | 323 | 1,495 | 1.00 | Reference |  |
| Presence | 49 | 172 | 1.65 | 1.20–2.26 | 0.002 |
| ***Alzheimer’s disease*** |  |  |  |  |  |
| Retinopathy |  |  |  |  |  |
| Absence | 232 | 1,495 | 1.00 | Reference |  |
| Presence | 30 | 172 | 1.52 | 1.02–2.26 | 0.04 |
| ***Vascular dementia*** |  |  |  |  |  |
| Retinopathy |  |  |  |  |  |
| Absence | 55 | 1,495 | 1.00 | Reference |  |
| Presence | 11 | 172 | 1.82 | 0.92–3.59 | 0.08 |
| Abbreviations: CI, confidence interval; HR, hazard ratio.  ^a)^ Adjusted for age, sex, education, systolic blood pressure, use of antihypertensive agents, diabetes mellitus, serum total cholesterol, body mass index, history of stroke, smoking habits, alcohol intake, and regular exercise. | | | | | |

| **eTable 3.　Baseline characteristics of participants according to the presence and absence of retinopathy among individuals without hypertension nor diabetes: the Hisayama Study, 2007.** | | | |
| --- | --- | --- | --- |
| Variable | Without hypertension or diabetes mellitus (n=577) | | *p* value |
|  | Retinopathy | |  |
|  | Absence  (n = 556) | Presence  (n = 21) |  |
| Age, mean (SD), y | 69.4 (7.2) | 72.4 (10.0) | 0.06 |
| Male, % | 39.2 | 47.6 | 0.44 |
| Education ≤ 9 years, % | 36.7 | 38.1 | 0.90 |
| Systolic blood pressure, mean (SD), mmHg | 122 (11) | 124 (9) | 0.41 |
| Diastolic blood pressure, mean (SD), mmHg | 74 (7) | 74 (7) | 0.95 |
| Fasting blood glucose, mean (SD), mmHg | 97.1 (8.1) | 95.0 (7.7) | 0.25 |
| Casual or 2-h postload glucose, mean (SD), mmHg | 121.6 (26.1) | 121.3 (25.0) | 0.45 |
| Serum total cholesterol, mean (SD), mmol/L | 5.56 (0.96) | 5.40 (1.03) | 0.49 |
| Body mass index, mean (SD), kg/m^2^ | 22.1 (3.0) | 21.3 (1.6) | 0.24 |
| History of stroke, % | 2.2 | 0 | - |
| Smoking habits, % | 14.9 | 19.1 | 0.61 |
| Alcohol intake, % | 38.9 | 38.1 | 0.94 |
| Regular exercise ≥ 3 times/w, % | 15.1 | 9.5 | 0.49 |
| Abbreviations: SD, standard deviation.  All values are given as the mean or as a percentage. | | | |
